# Optical and Photocatalytic Properties of Br-Doped BiOCl Nanosheets with Rich Oxygen Vacancies and Dominating {001} Facets

**DOI:** 10.3390/nano12142423

**Published:** 2022-07-15

**Authors:** Qian Zhang, Wuyang Nie, Tian Hou, Hao Shen, Qiang Li, Chongshang Guan, Libing Duan, Xiaoru Zhao

**Affiliations:** 1 MOE Key Laboratory of Material Physics and Chemistry under Extraordinary Conditions, Northwestern Polytechnical University, Xi’an 710072, China; 2018100283zhangqian@mail.nwpu.edu.cn (Q.Z.); nwy@mail.nwpu.edu.cn (W.N.); houtian@nwpu.edu.cn (T.H.); qli029@163.com (Q.L.); csguan@mail.nwpu.edu.cn (C.G.); lbduan@nwpu.edu.cn (L.D.); 2 Shaanxi Key Laboratory of Condensed Matter Structures and Properties, Northwestern Polytechnical University, Xi’an 710072, China; 3 Department of Applied Physics, School of Physical Science and Technology, Northwestern Polytechnical University, Xi’an 710072, China; 4 Department of Applied Physics, Chang’an University, Xi’an 710064, China; chshen@126.com

**Keywords:** BiOCl nanosheets, Br doping, surface facet, oxygen vacancies, photocatalytic performance

## Abstract

Crystal facet engineering and nonmetal doping are regarded as effective strategies for improving the separation of charge carriers and photocatalytic activity of semiconductor photocatalysts. In this paper, we developed a facial method for fabricating oxygen-deficient Br-doped BiOCl nanosheets with dominating {001} facets through a traditional hydrothermal reaction and explored the impact of the Br doping and specific facets on carrier separation and photocatalytic performance. The morphologies, structures, and optical and photocatalytic properties of the obtained products were characterized systematically. The BiOCl samples prepared by the hydrothermal reaction exhibited square-like shapes with dominating {001} facets. Photodeposition results indicated that photoinduced electrons preferred to transfer to {001} facets because of the strong internal static electric fields in BiOCl nanosheets with dominating {001} facets. Br doping not only contributed to the formation of impurity energy levels that could promote light absorption but introduced a large number of surface oxygen vacancies (V_O_) in BiOCl photocatalysts, which was beneficial for photocatalytic performance. Moreover, the photocatalytic activities of these products under visible light were tested by degradation of rhodamine B (RhB). Because of the synergistic effect of the dominating {001} facets, Br doping, and rich V_O_, oxygen-deficient Br-doped BiOCl nanosheets exhibited improved carrier separation, visible light absorption, and photocatalytic efficiency.

## 1. Introduction

Water pollution has become a crucial issue as a result of excessive emissions of various organic pollutants that seriously impede the existence and development of humanity [1,2,3]. Therefore, to address the issue of water contamination, effective and environmentally friendly technology must be developed. During the last decades, people have developed many semiconductor materials (such as ZnO, TiO_2_, CeO_2_, and MoS_2_) as efficient photocatalysts to purify wastewater [4,5,6,7]. In the midst of these semiconductor materials, BiOCl, composed of [Bi_2_O_2_]^2+^ layers interleaved with double Cl^-^ layers, has received extensive attention and fascination owing to its nontoxicity, environmental stability, low cost, open crystalline structure, and indirect-transition bandgap [8,9,10].

Nevertheless, because of the large bandgap energy and short lifespan of photoinduced carriers, BiOCl exhibits inferior visible-light photocatalytic performance, which limits its applications in energy conversation [11,12]. To further enhance the photocatalytic efficiency of BiOCl, numerous methods have been performed to promote the absorption efficiency of visible light and prolong the lifespan of charge carriers, such as metal or nonmetal element doping and constructing heterojunctions with narrow-bandgap semiconductors or carbon materials [13,14,15,16]. Crystal facet engineering of semiconductors has received much attention because photoinduced electrons and holes might be steered to different crystal facets, and this spatial separation of charge carriers might improve the photocatalytic activity of BiOCl [17,18]. Previous research clarified that BiOCl nanosheets with dominating {001} facets showed superior photocatalytic efficiency owing to the assistance of internal static electric fields perpendicular along {001} facets, which contributed to spatial separation of charge carriers [19,20]. Nonetheless, because the spatial separation efficiency of charge carriers in BiOCl photocatalysts with specific crystal facets is still limited, it is necessary to promote the separation of photoexcited carriers to further improve photocatalysis [21]. Besides crystal facet engineering, other methods have been tried to improve the photodegradation efficiency of BiOCl, such as element doping (including defects and metal and nonmetal elements) [22]. It has been proven that doping nonmetal elements into semiconductor materials generates impurity energy levels above the valence band (VB) to further promote optical property of semiconductor, which could effectively enhance the efficiency of carrier separation [23]. Liu et al. [24] prepared iodine-doped BiOCl nanosheets surrounded by {001} and {110} crystal facets that possessed superior photodegradation efficiency. Moreover, it is generally accepted that oxygen vacancies (V_O_) in semiconductors play a significant role in regulating their optical and photocatalytic properties. Previous reports showed that introduction of moderate V_O_ could significantly improve the photocatalytic efficiency of nanomaterials [25,26]. The V_O_ could not only act as reactive sites in BiOCl for photocatalytic activity but trap photoexcited electrons and inhibit charge carrier recombination [27,28]. Several strategies, such as high-energy ball milling and chemical reduction methods, have been used to promote the generation of V_O_ in semiconductor materials [29]. Yu et al. [30] prepared BiOCl microflowers with rich V_O_ via a facial one-pot solvothermal method, and the modified BiOCl exhibited improved photocatalytic efficiency. Song et al. [31] adopted a facile hydrolysis approach to acquire oxygen-deficient BiOCl nanosheets in order to achieve improved photocatalytic efficiency.

Remarkable progress on BiOCl photocatalysts have been made in the past several years through crystal facet engineering, element doping, and constructing heterojunctions with narrow-bandgap semiconductors. However, the challenge still exists to enhance the photodegradation efficiency of BiOCl for achieving practical applications. Recent reports showed that a BiOCl photocatalyst with rich V_O_ possessed enhanced photocatalytic activity and a prolonged lifespan of charge carriers. However, they did not consider the influence of specific facets. Considering previous reports, BiOCl with dominating {001} facets simultaneously modified with Br and V_O_ would be expected to perform well in terms of photodegradation efficiency and visible light absorption. However, little work has focused on the synergistic effect of Br doping and V_O_ in BiOCl with dominating {001} facets photocatalyst. Moreover, few papers have discussed the changes in the V_O_ in BiOCl crystals caused by Br doping in detail. The mechanism of facet-dependent photocatalytic properties is still unclear.

In this paper, oxygen-deficient Br-doped BiOCl nanosheets with dominating {001} facets were successfully fabricated by a traditional hydrothermal method. The morphologies, structures, and chemical and photoelectric properties of the products were analyzed in detail. The degradation efficiency of these products under visible light was tested by photodegradation of RhB. As for Br-doped BiOCl nanosheets, Br doping not only contributed to the formation of impurity energy levels that could promote the light absorption but introduced rich V_O_ on the surface of BiOCl photocatalysts, which was beneficial for photocatalytic performance. In addition, BiOCl nanosheets with dominating {001} facets could transfer the photoinduced electrons to {001} crystal facets and avoid the fast recombination of carriers. Square-like shapes with cheap thickness could also decrease the diffusing distance over which photoinduced electrons migrate to the surface of the photocatalysts. As expected, the prepared oxygen-deficient Br-doped BiOCl photocatalysts exhibited improved degradation efficiency under visible light for RhB aqueous solution degradation.

## 2. Materials and Methods

***Materials*:** Sodium chloride (NaCl) and bismuth nitrate pentahydrate (Bi(NO_3_)_3_∙5H_2_O) were obtained from Tianjin Damao Chemical Reagent Co., Ltd. (Tianjin, China). Mannitol (CH_2_(OH)(CHOH)_4_CH_2_OH) was received from Tianjin Shenao Chemical Reagent Co., Ltd. (Tianjin, China). Potassium bromide (KBr) was obtained from Shanghai Aladdin Biochemical Technology Co., Ltd. (Shanghai, China). Tetracycline hydrochloride (C_22_H_24_N_2_O_8_∙HCl) was obtained from Shanghai Macklin Biochemical Co., Ltd. (Shanghai, China). Rhodamine B (C_28_H_31_ClN_2_O_3_) was received from Tianjin Fuchen Chemical Reagent Co., Ltd. (Tianjin, China). Methyl orange (C_14_H_14_N_3_NaO_3_S) was obtained from Sinopharm Chemical Reagent Co., Ltd. (Shanghai, China).

***Preparation of BiOCl nanosheets*:** BiOCl nanosheets were synthesized via a facial hydrothermal reaction. In detail, 0.490 g Bi(NO_3_)_3_∙5H_2_O, was put into 25.0 mL of 0.1 M mannitol aqueous solution with magnetic stirring for 30 min (marked as solution A). Next, 1.762 g of NaCl was put into 6 mL water with magnetic stirring for 30 min (marked as solution B) and then mixed with solution A. The mixture was loaded into a 50 mL Teflon-lined autoclave and held at 160 °C for 3 h. After that, BiOCl nanosheets were obtained via centrifugation, washed three times, and dried at 60 °C for 6 h.

***Preparation of Br-doped BiOCl nanosheets*:** Br-doped BiOCl nanosheets were produced using a similar method as described above. In brief, after solution A was prepared, stoichiometric amounts of NaCl and KBr with a total concentration of 5.0 M were dissolved into 6 mL distilled water and then added into solution A with magnetic stirring for 30 min. The other steps were identical to those of the method for preparing BiOCl nanosheets. BiOCl nanosheets with Br−doped contents of 0, 0.5, 1, and 2 at. % were denoted as BOC, Br−BOC−0.5, Br−BOC−1, and Br−BOC−2, respectively.

***Characterization*:** The crystal structures of the as-prepared photocatalysts were studied by X-ray diffraction (XRD) patterns on a PANalytical X’pert MPD PRO (Cu *Kα* radiation). The microstructure and morphologies of the obtained products were explored via transmission electron microscopy (TEM, Tecnai F30 G2, FEI Company, Hillsboro, OR, USA) and scanning electron microscopy (SEM, JSM-7000F, JEOL, Tokyo, Japan). Electron spin resonance (ESR) spectra were obtained via a Bruker EMXPLUS EPR spectrometer (Bruker, Billerica, MA, USA) to analyze the oxygen vacancy defects of the obtained photocatalysts. X-ray photoelectron spectroscopy (XPS) spectra were obtained via a Kratos AXIS Ultra DLD spectrometer (Kratos, Manchester, UN) to study the element composition of products. The light absorption abilities of the samples were studied through a PE Lambda 950 spectrophotometer (PerkinElmer, Waltham, MA, USA) by UV–vis diffuse reflectance spectroscopy (DRS). Photoluminescence (PL) spectra were obtained with a Gangdong F-320 photoluminescence spectrophotometer (Tianjin Gangdong, Tianjin, China) at an excitation wavelength of 275 nm.

***Photocatalytic activity test*:** The photocatalytic test was carried out through the decomposition of RhB with a 500 W Xeon-lamp and a 420 nm cutoff filter. In detail, 10 mg samples were put into RhB solution (50 mL, 20 mg/L) with stirring for 1 h before light on to obtain the adsorption/desorption balance between the photocatalysts and the RhB. At 20 min intervals, 3 mL mixed solution was extracted, and photocatalysts were removed by centrifugation immediately. By using a UV–vis spectrophotometer to track changes in dye concentrations, the removal ratio of RhB was determined through the equation E = C/C_0_, where C_0_ and C represented the concentration after adsorption equilibrium and the corresponding concentration in real time, respectively.

***Photodeposition tests:*** The photodeposition experiment was carried out as follows. Photocatalyst (50 mg) and H_2_PtCl_6_∙6H_2_O solution (20 mg/L, 150 μL) were mixed with 50 mL deionized water. Then, the solution was irradiated under mercury lamp (500 W) with continuous stirring. After photodeposition for 30 min, the photocatalysts were obtained after centrifugation and washed before being dried at 60 °C for 6 h.

## 3. Results and Discussion

**Figure 1:**
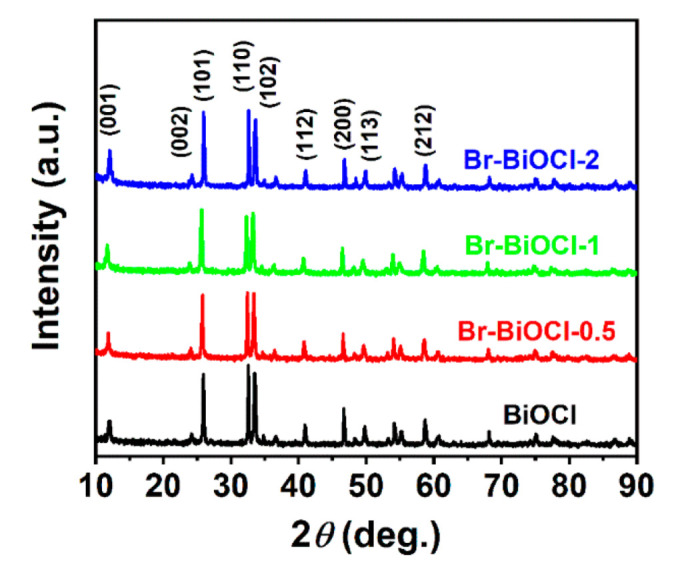
XRD pattern of the as-prepared samples.

The phases and crystal structures of the obtained products were studied by XRD. As displayed in Figure 1, all of the obtained products possessed superior crystallinity, and the peaks located at 11.98°, 24.15°, 26.05°, 32.56°, 33.60°, 40.95°, 46.72°, 49.76°, and 58.68° could be indexed to the tetrahedral BOC (JCPDS 06-0249) [32,33]. There were no distinctive peaks from other phases or contaminants, suggesting the fine purity of the products.

**Figure 2:**
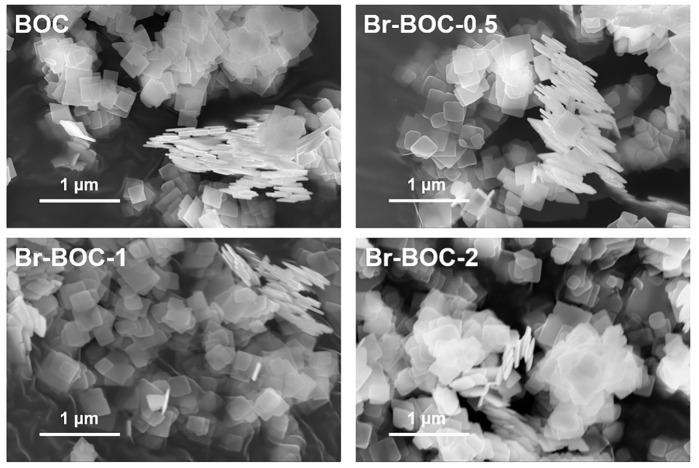
SEM patterns of BOC, Br−BOC−0.5, Br−BOC−1, and Br−BOC−2.

**Figure 3:**
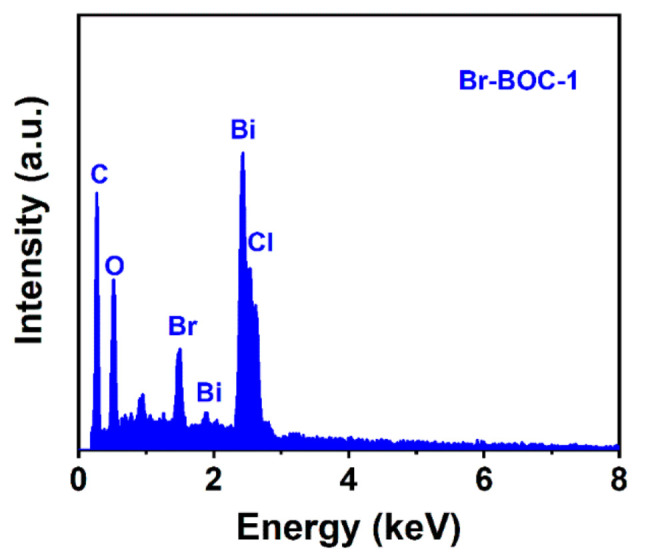
EDS analyses of Br−BOC−1.

SEM was used to study the surface morphologies of BOC, Br−BOC−0.5, Br−BOC−1, and Br−BOC−2. BOC displayed square-like shapes with a width of 150–275 nm and a thickness of 28–47 nm, as shown in Figure 2. The size and shape of BiOCl was maintained after the introduction of moderate Br, indicating that Br doping did not impede the growth of BiOCl nanosheets. Energy disperse spectroscopy (EDS) measurement was performed to study the composition information of the obtained products. As shown in Figure 3, the EDS results proved the existence of Bi, O, Cl, and Br, which suggested that Br was doped into the BiOCl nanosheets. The BET surface areas of BOC, Br−BOC−0.5, Br−BOC−1, and Br−BOC−2 were 11.18 m^2^g^−1^, 12.45 m^2^g^−1^, 12.85 m^2^g^−1^, and 12.67 m^2^g^−1^, respectively. The BET special surface area of BiOCl showed a slight increase after Br-doping, suggesting that the BET special surface area in this work was not a major factor affecting the degradation efficiency. The BET surface areas for all samples were tabulated in Table 1.

**Figure 4:**
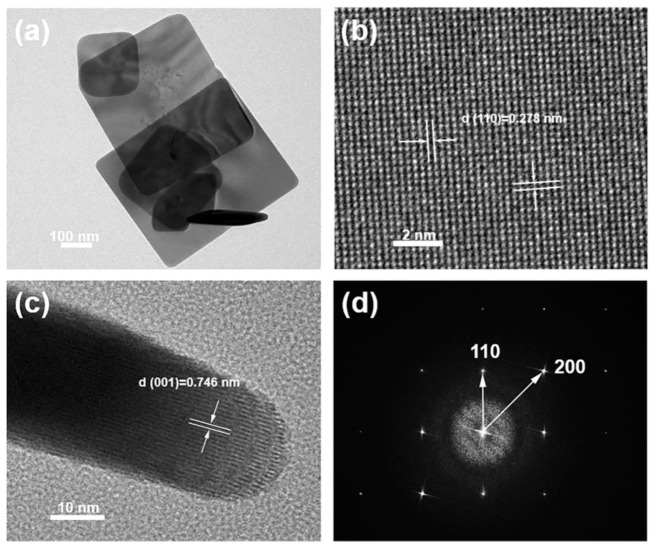
(**a**) TEM image of Br−BOC−1, (**b**) HRTEM image taken from the top view of Br−BOC−1, (**c**) HRTEM image taken from the side view of Br−BOC−1, (**d**) SAED pattern taken from the top view of Br−BOC−1.

TEM and high-resolution TEM (HRTEM) were performed to study the structural features and exposed crystal facets of Br−BOC−1. A TEM image, as displayed in Figure 4a, clearly revealed its square-like patterns once more. Figure 4b shows an HRTEM image from the top view of a nanosheet. The interplanar spacing was measured as 0.278 nm, which corresponded to the (110) planes. The HRTEM image of the side view exhibited clear lattice fringes. The interplanar spacing was measured as 0.746 nm, as displayed in Figure 4c, which was matched with the (001) planes of the BiOCl crystal. Figure 4d shows the selected-area electron diffraction (SAED) pattern of the top view, which proved the single-crystalline characteristic of Br−BOC−1. The angle of the two marked planes was 45°, which corresponded to the theoretical value of the angle between the (110) and (200) planes [34,35]. Based on the above analysis of Br−BOC−1, the top and bottom surfaces of Br−BOC−1 were surrounded by {001} facets, and the four lateral surfaces were enclosed by {110} facets.

**Figure 5:**
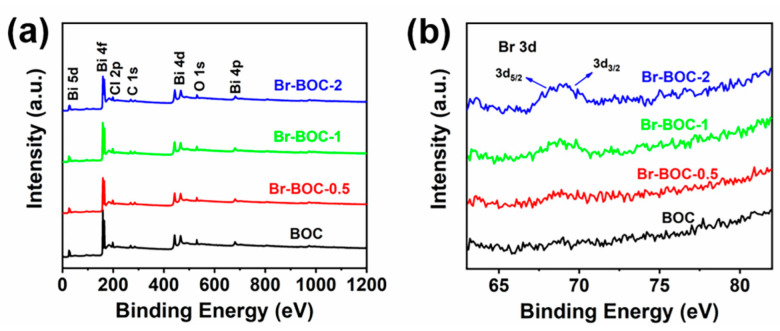
(**a**) XPS survey spectra and (**b**) Br 3d high-resolution XPS spectra of as-prepared samples.

The surface chemical compositions of the obtained products were investigated by XPS. The full-scale XPS patterns of the products, BOC, Br−BOC−0.5, Br−BOC−1 and Br−BOC−2, are displayed in Figure 5a. Peaks corresponding to Bi 5d, Bi 4f, Cl 2p, C 1s, Bi 4d, O 1s, and Bi 4p were found in all samples. Peaks corresponding to Br did not appear in this pattern, which might be attributable to the low doping ratio. Br 3d high-resolution XPS spectra of all products were obtained to confirm the incorporation of the Br atom into Br−BOC−0.5, Br−BOC−1, and Br−BOC−2. As exhibited in Figure 5b, compared with BOC, a new broad peak appeared around 69 eV for Br−BOC−0.5, Br−BOC−1, and Br−BOC−2, which could have been associated with the Br 3d_5/2_ and Br 3d_3/2_ peaks [36]. The intensity of these peaks increased as the doping concentration of the Br atom increased, which further proved that Br was doped into BiOCl.

**Figure 6:**
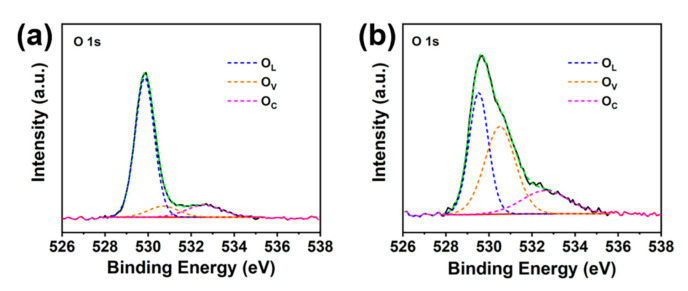

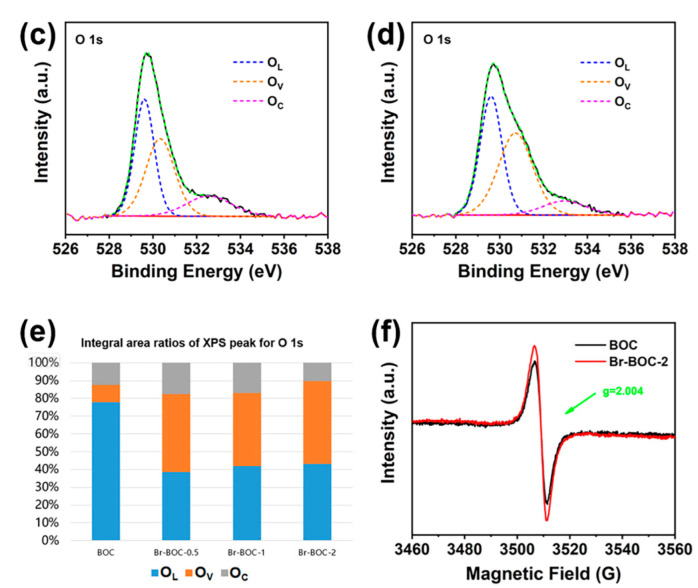
O 1s XPS spectra of (**a**) BOC, (**b**) Br−BOC−0.5, (**c**) Br−BOC−1, and (**d**) Br−BOC−2; (**e**) the integral area ratios of the XPS peak for O 1s, (**f**) ESR spectra of BOC and Br−BOC−1.

To acquire more information on surface defects in the BOC, Br−BOC−0.5, Br−BOC−1, and Br−BOC−2, the XPS spectra for O 1s were obtained, as exhibited in Figure 6a–d. The O 1s peak could be divided into three distinct peaks. The highest peak, at 530.1 eV, could be attributed to the lattice oxygen anions (O_L_) in the samples. The middle peak, at 531.4 eV, was related to the surface oxygen vacancies (O_V_) in the BiOCl nanosheets. The peak at 532.3 eV could be ascribed to the weakly bound oxygen or chemisorbed oxygen (O_C_) in the BiOCl [36]. Therefore, the change in the O_V_ peak intensity was related to the concentration of V_O_ in the products. The relative O_V_/O_T_ (O_T_ = O_L_ + O_V_ + O_C_) ratios for all products were obtained through the ratios of the areas of the peaks. These were used to assess the changes in V_O_ concentration and are listed in Figure 6e. Compared with the BOC nanosheets, the Br-doped BOC samples possessed sharply increased concentrations of V_O_. When the concentration of Br doping was high, the concentration of intrinsic oxygen vacancies might have become lower compared with the concentration of extrinsic oxygen vacancies. This might be the reason why increased concentrations of doped Br did not increase the ratio of oxygen vacancies. ESR spectroscopy was carried out to confirm the variation in V_O_. Figure 6f shows the ESR spectra of BOC and Br−BOC−1. An obvious signal (g = 2.004) was found that could be ascribed to the oxygen vacancy of the bound single electron (V_O_^+^) [37]. The signal intensity of Br−BOC−1 was much stronger than that of BOC, which further demonstrated that the Br-doped BiOCl nanosheets had richer surface V_O_ than the undoped BiOCl. This difference might give rise to different photodegradation efficiency in the photocatalytic process.

**Figure 7:**
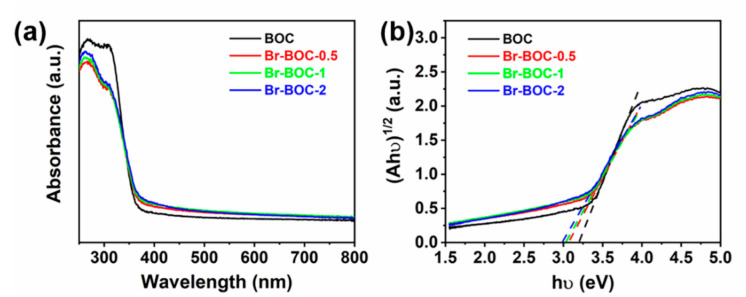
(**a**) UV–vis diffuse reflectance spectra of BOC, Br−BOC−0.5, Br−BOC−1, and Br−BOC−2 nanosheets; (**b**) the plot of transformed Kubelka-Munk function versus the energy of light.

The absorption properties of the BOC and Br-doped BOC nanosheets were explored by UV–vis diffuse reflectance spectroscopy (DRS). As exhibited in Figure 7a, the Br-doped BOC nanosheets displayed improved visible light absorption compared with the undoped BOC. The bandgap energy (Eg) was calculated through the equation:

αhυ = A(hυ − Eg)^n/2^

where α means the absorption coefficient of samples, A is a constant, h means the Planck constant, υ is the light frequency, and Eg is the bandgap energy [38]. Consequently, it was found that the Egs of these samples were 3.21, 3.06, 3.02, and 2.96 eV for BOC, Br−BOC−0.5, Br−BOC−1, and Br−BOC−2 respectively. It was clear that as the Br doping content increased, the bandgap energies of Br-doped BiOCl gradually decreased. Therefore, because of the reduced bandgap, the visible light absorption efficiency of the Br-doped BOC was enhanced. We propose that the impurity level caused by Br doping in the semiconductor was responsible for the photocatalytic activity of the samples, allowing the absorption of visible photons through a step-by-step mechanism.

**Figure 8:**
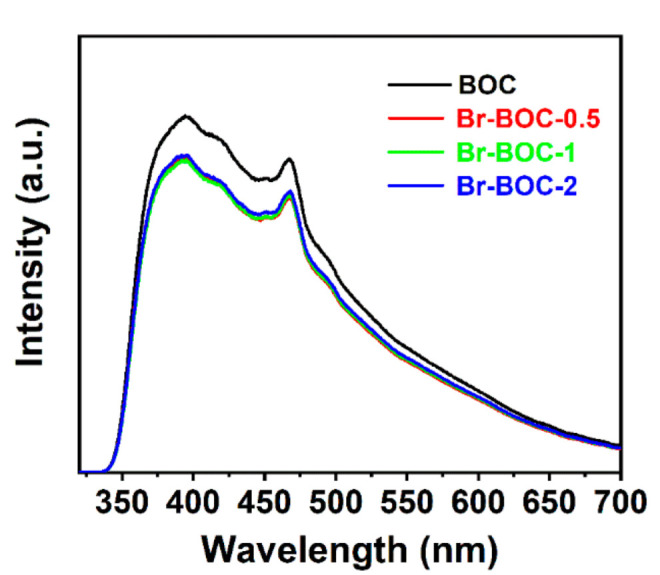
PL spectra of as-prepared BOC, Br−BOC−0.5, Br−BOC−1, and Br−BOC−2 nanosheets.

PL spectra were used to study the transformation and recombination of charge carriers. Figure 8 displays the PL spectra of the BOC and Br-doped BOC. The PL intensity was deemed to be associated with the lifespan of charge carriers, and higher PL emission peaks resulted from photoinduced carriers recombining more quickly [39]. The lower emission peak for the Br-doped BiOCl samples than for the BOC nanosheets indicated that the Br-doped BOC nanosheets possessed longer lifespans for their charge carriers, which could be ascribed to the introduction of V_O_ that could trap photoinduced electrons and thereby prolong the lifespan of the charge carriers.

**Figure 9:**
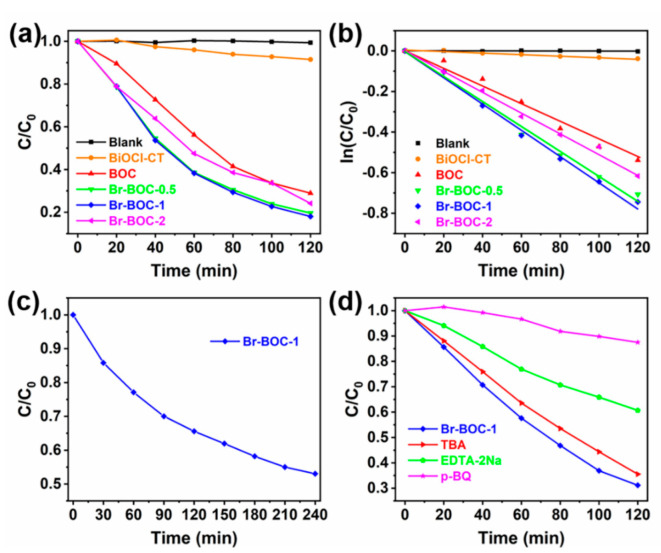
(**a**) Photocatalytic degradation of RhB by BOC, Br−BOC−0.5, Br−BOC−1, and Br−BOC−2; (**b**) the corresponding kinetic curves; (**c**) photocatalytic degradation of tetracycline hydrochloride by Br−BOC−1; (**d**) active species trapping experiments for Br−BOC−1 with different scavengers under visible light.

The degradation efficiencies of BOC and Br-doped BOC nanosheets under visible light were explored according to the removal ratio of RhB, as exhibited in Figure 9a, where C_0_ means the RhB content before light on and C is the corresponding concentration in real time. An adsorption–desorption balance was carried out before light on. Considering possible self-photodegradation for RhB, a blank experiment in the absence of photocatalysts was carried out as a control line, as shown in Figure 9a. The concentration of RhB did not decline without photocatalysts, which meant that self-photodegradation for RhB was almost neglectable. All samples displayed enhanced degradation efficiency compared with the commercial BiOCl (BiOCl−CT), indicating that BiOCl nanomaterials with exposed {001} facets possessed improved degradation efficiency. After Br element doping, the degradation efficiency of Br-doped BiOCl obviously improved. The curves of Br−BOC−0.5, Br−BOC−1, and Br−BOC−2 slightly intermixed, which might have been attributable to the similar Br doping concentrations. Br−BOC−1 exhibited the highest degradation efficiency, and the concentration of RhB was reduced to 18% after 120 min under visible light photoirradiation. The degradation efficiency of the obtained products was evaluated quantitatively through a pseudo-first-order kinetics equation:

In(C/C_0_) = −kt

where C_0_ and C represent the initial RhB content and the content after the different irradiation times, respectively [40]. The corresponding kinetic curves are exhibited in Figure 9b, and Br−BOC−1 had a much higher apparent rate constant k (k = 0.00656 min^−1^) than BOC (k = 0.00445 min^−1^). To rule out the influence of the sensitization of the dye, the degradation of tetracycline hydrochloride (colorless compound, 20 mg/L) under visible light irradiation was performed. As displayed in Figure 9c, Br−BOC−1 presented excellent degradation efficiency for tetracycline hydrochloride under visible light. Photodegradation experiments with various scavengers were performed to further analyze the RhB removal mechanism of the samples. In order to detect O_2_, OH, and h^+^, p-benzoquinone (p-BQ), tert-butyl alcohol (TBA), and disodium ethylenediaminetetraacetate (EDTA-2Na) were dissolved to the reaction solution, respectively. As displayed in Figure 9d, there existed obvious decreases in degradation efficiency in the presence of EDTA-2Na and p-BQ, which meat that h^+^ and ˙O_2_^−^ were the main active species that contributed to the photocatalytic performance. To explore the adsorption ability of the samples, RhB adsorption experiments were carried out. The adsorption rates of BOC, Br−BOC−0.5, Br−BOC−1, and Br−BOC−2 were calculated as 37%, 41%, 46%, and 40%. Br-doped BOC possessed higher adsorption ability for RhB, which could further promote photocatalytic activity. The dye removal efficiency was calculated through the following equation:

Dye removal (%) = ((C_0_ − C)/C_0_) × 100%

where C_0_ and C represent the initial RhB content and that after the different irradiation times, respectively. As shown in Figure S1, Br-doped BiOCl exhibited improved RhB removal efficiency, and Br−BOC−1 displayed the highest dye removal efficiency of 90%.

**Figure 10:**
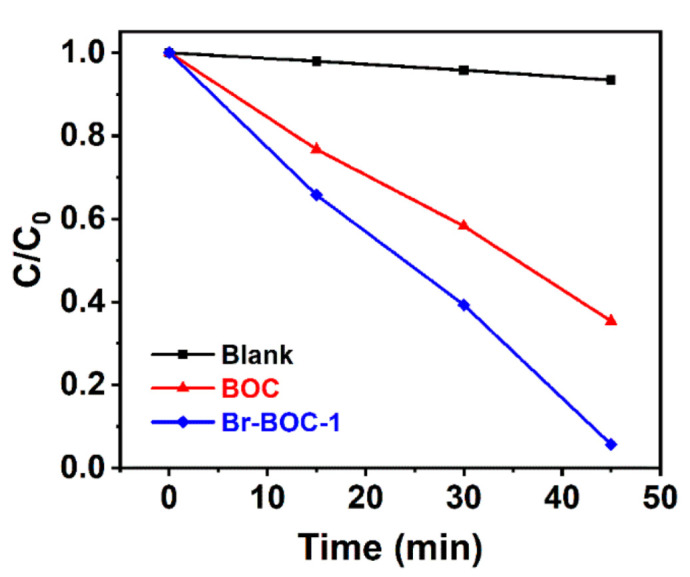
Photocatalytic degradation of MO by BOC and Br−BOC−1.

To eliminate the influence of improved photoabsorption caused by doping, photocatalysis experiments were carried out under UV light to degrade methyl orange (MO, 20 mg/L) to further analyze the utilization rate of photoinduced carriers in the process of the photocatalytic reaction. Considering the serious self-photodegradation for RhB under UV light, MO was chosen as a target material. An adsorption–desorption equilibrium was performed before light on. As displayed in Figure 10, Br−BOC−1 possessed enhanced degradation efficiency compared with BOC, and the concentration of MO was reduced to 6% in the presence of Br−BOC−1 after 45 min under UV light, which might be ascribed to the formation of rich V_O_ on the surface of Br−BOC−1. V_O_ could not only capture photoinduced carriers and prolong their lifespan but serve as active sites to accelerate the photodegradation process. These results also demonstrated that Br−BOC−1 photocatalysts still had superior degradation efficiency under UV light. Figure S2 exhibited the adsorption and photodegradation curves of BOC and Br−BOC−1 under UV light, where 95% of MO was removed by Br−BOC−1 after 45 min irradiation.

**Figure 11:**
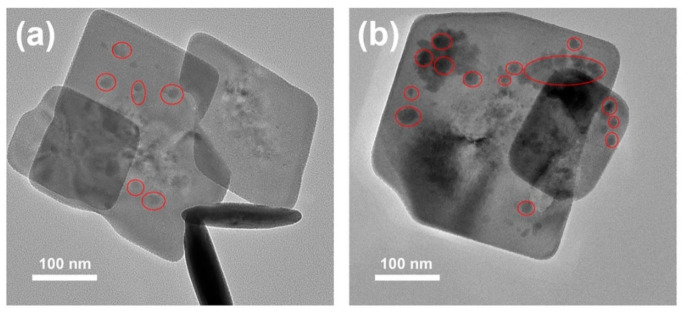
TEM images of BOC (**a**) and Br−BOC−1 (**b**) with Pt photodeposited under UV light irradiation.

For the building of an efficient solar energy conversion system, a deep understanding of carrier separation and transfer inside semiconductors is crucial. Using H_2_PtCl_6_ as a precursor, a photoreduction deposition experiment on Br−BOC−1 was performed. As exhibited in Figure 11b, a TEM picture revealed that Pt particles tended to photodeposit on the {001} facets. In other word, the photoexcited electrons preferred to transfer and accumulate on the {001} facets of Br−BOC−1 to react with Pt^+^. Moreover, to verify the improved spatial separation of charge carriers, a photodeposition experiment on BOC was performed under the same conditions. As displayed in Figure 11a, the reactive sites of photoreduction of the BOC nanosheets were consistent with those of the doped BOC nanosheets. However, compared with Br−BOC−1, fewer Pt particles were generated on the {001} facets. These results unambiguously demonstrated that photoexcited electrons tended to migrate to the {001} facets of BOC with the help of an internal electric field, which contributed to carrier separation and transfer, resulting in reduction reactions. Enhanced separation of photoexcited electrons and holes of BiOCl nanosheets was achieved by comodification with Br and oxygen vacancies.

**Scheme 1:**
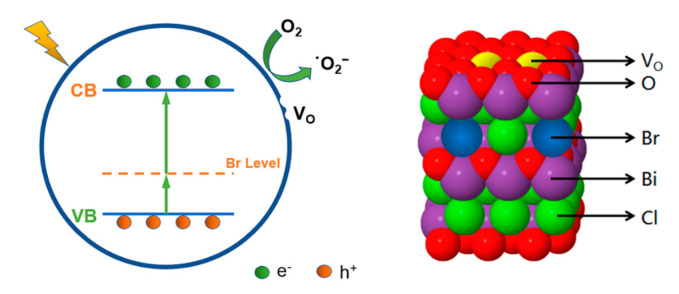
Band structure of the Br−doped BOC and schematic of the separation and transfer of charge charges in Br−doped BOC nanosheets.

In Scheme 1, we propose a possible mechanism for RhB photodegradation under visible light over Br-doped BiOCl nanosheets based on the aforementioned analysis. In the proposed mechanism, because of the irradiation of light, electrons would transfer from the valence band (VB) to the conduction band (CB) of BiOCl with the assistance of impurity energy levels between the VB and CB introduced by Br doping, and holes would be left on the VB. With the aid of an internal electric field, photoinduced electrons would accumulate on the surface of {001} crystal facets and avoid fast recombination. In addition, rich V_O_ on the surface of Br-doped BiOCl would not only capture photoinduced electrons but act as reactive sites that further promote the efficiency enhancement of photocatalysis. In summary, the synergistic effect of the surface facets and comodification with Br and V_O_ would enhance the photodegradation efficiency of Br-doped BiOCl.

## 4. Conclusions

Oxygen-deficient Br-doped BiOCl nanosheets with dominating {001} facets were successfully fabricated by a hydrothermal reaction. In the process of synthesis, because of the larger ionic radius of Br^−^ than Cl^−^, introduction of Br led to lattice distortion in the BOC, which contributed to the formation of rich V_O_. BiOCl nanosheets with dominating {001} facets promoted the transfer of photoexcited electrons to {001} crystal facets with the help of an internal electric field and prevented fast recombination of photoinduced carriers. Br doping introduced impurity energy levels above the VB and promoted visible light absorption. Moreover, V_O_ on the surface of the BOC could not only capture photoinduced electrons but serve as the sites for redox reactions. Because of the synergistic effect of surface facets and comodification with Br and V_O_, Br-doped BiOCl nanosheets showed improved photocatalytic activity. Br−BOC−1 exhibited the highest degradation efficiency, and the concentration of RhB was reduced to 18% after 120 min under visible light photoirradiation. In addition, 95% of MO was removed after 45 min in the presence of Br−BOC−1, which proved that Br−BOC−1 also possessed excellent degradation efficiency under UV light. This work may provide a promising opportunity to construct new photocatalysts with efficient carrier separation and superior photoabsorption.

## Figures and Tables

**Table 1:** Summary of Eg and BET data for all samples.

	BOC	Br−BOC−0.5	Br−BOC−1	Br−BOC−2
Eg (eV)	3.21	3.06	3.02	2.96
SBET (m^2^g^−1^)	11.18	12.45	12.85	12.67

## Data Availability

The date presented in this study are available on reasonable request from the corresponding author.

## Supplementary Materials

The following supporting information can be downloaded at: https://www.mdpi.com/article/10.3390/nano12142423/s1, Figure S1: Adsorption and photodegradation curves of RhB versus time under dark and visible light illumination; Figure S2: Adsorption and photodegradation curves of MO versus time under dark and UV light illumination.
