# Supplementary material for: Optical and Photocatalytic Properties of Br-Doped BiOCl Nanosheets with Rich Oxygen Vacancies and Dominating {001} Facets
Source: Nanomaterials (Basel). 2022 Jul 15;12(14):2423. doi: 10.3390/nano12142423 (PMC9318533; doi:10.3390/nano12142423)
Supplement: Supplementary file 1 [file nanomaterials-12-02423-s001.zip › nanomaterials-1797876-supplementary.pdf]

Supplementary Materials

Optical and Photocatalytic Properties of Br-Doped BiOCl Nanosheets with Rich Oxygen Vacancies and Dominating {001} Facets

Qian Zhang [1,2,3], Wuyang Nie [1,2,3], Tian Hou [1,2,3], Hao Shen [4], Qiang Li [1,2,3], Chongshang Guan [1,2,3], Libing Duan [1,2,3] and Xiaoru Zhao [1,2,3,*]

[1] MOE Key Laboratory of Material Physics and Chemistry under Extraordinary Conditions, Northwestern Polytechnical University, Xi'an 710072, China; 2018100283zhangqian@mail.nwpu.edu.cn (Q.Z.); nwy@mail.nwpu.edu.cn (W.N.); houtian@nwpu.edu.cn (T.H.); qli029@163.com (Q.L.); csguan@mail.nwpu.edu.cn (C.G.); lbduan@nwpu.edu.cn (L.D.)

[2] Shaanxi Key Laboratory of Condensed Matter Structures and Properties, Northwestern Polytechnical University, Xi'an 710072, China

[3] Department of Applied Physics, School of Physical Science and Technology, Northwestern Polytechnical University, Xi'an 710072, China

[4] Department of Applied Physics, Chang'an University, Xi'an 710064, China; chshen@126.com

* Correspondence: xrzhao@nwpu.edu.cn

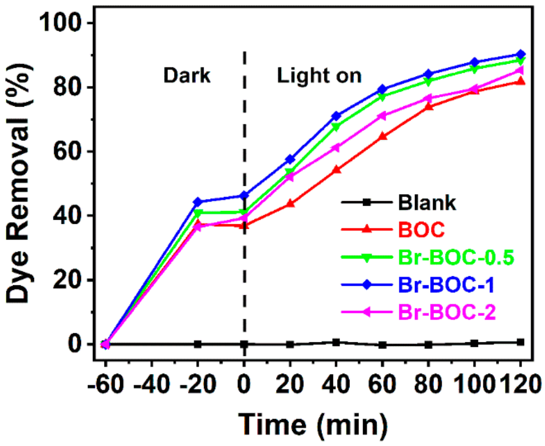

Figure S1. Adsorption and photodegradation curves of RhB versus time under dark and visible light illumination.

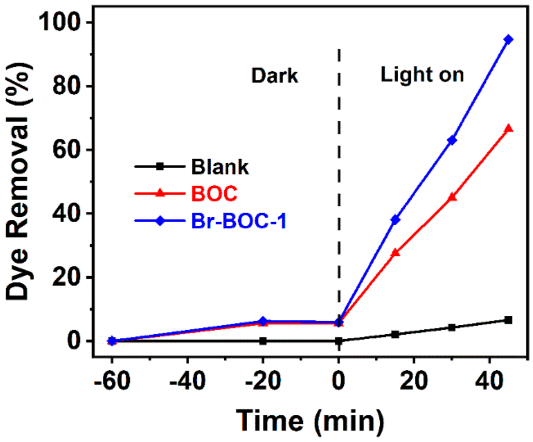

Figure S2. Adsorption and photodegradation curves of MO versus time under dark and UV light illumination.
